# Supplementary material for: In vivo engineered extracellular matrix scaffolds with instructive niches for oriented tissue regeneration
Source: Nat Commun. 2019 Oct 11;10:4620. doi: 10.1038/s41467-019-12545-3 (PMC6789018; doi:10.1038/s41467-019-12545-3)
Supplement: Supplementary file 1 — Supplementary Information [file 41467_2019_12545_MOESM1_ESM.pdf]

Supplementary Information

In vivo engineered extracellular matrix scaffolds with instructive niches for oriented tissue regeneration

Meifeng Zhu, Wen Li et al.

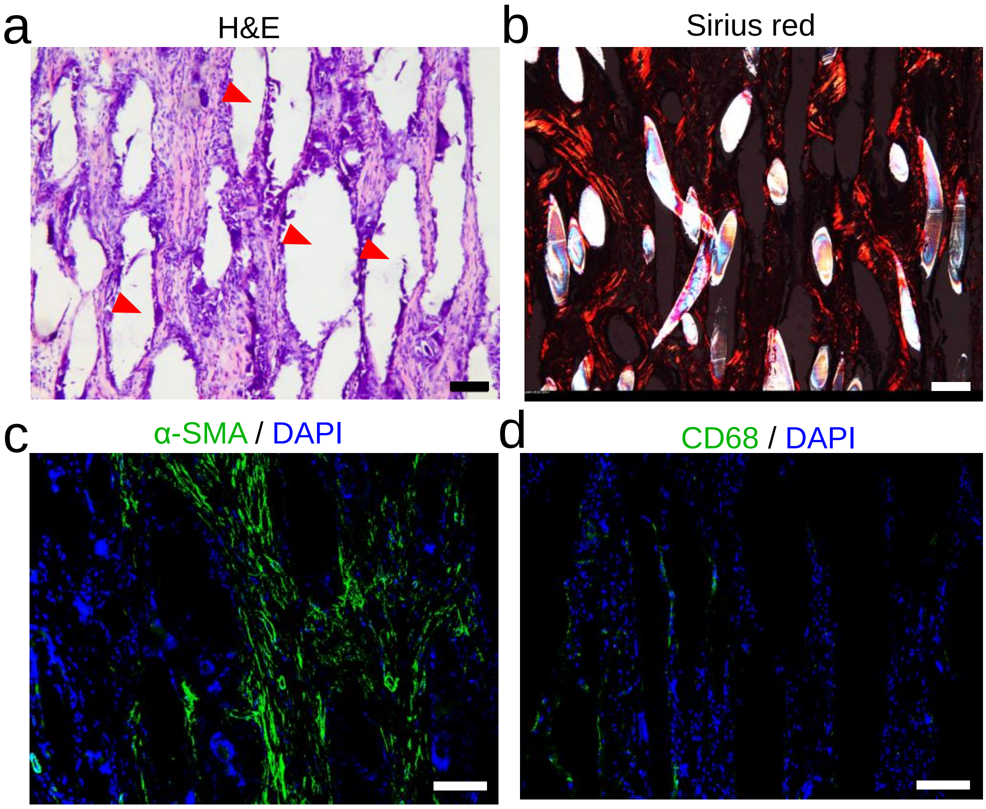

Supplementary Figure 1 Cellularization of PCL templates after 4-week subcutaneous implantation. **a**, H&E staining of the cross-sections of cellularized PCL templates showed that the void gaps among the aligned microfibers were fully occupied by cells and cell-secreted ECM. Red triangles indicate the position of the PCL fibers. **b**, Sirius red staining of the cross-sections showed the distribution and arrangement of collagen fibers within cellularized PCL templates. PCL fibers are white. **c**, **d**, Immunofluorescence staining of the cross-sections showed that cells within the void gaps of aligned microfibers were mostly positive for α-SMA with only a few positive for CD68. Scale bars: 100 μm.

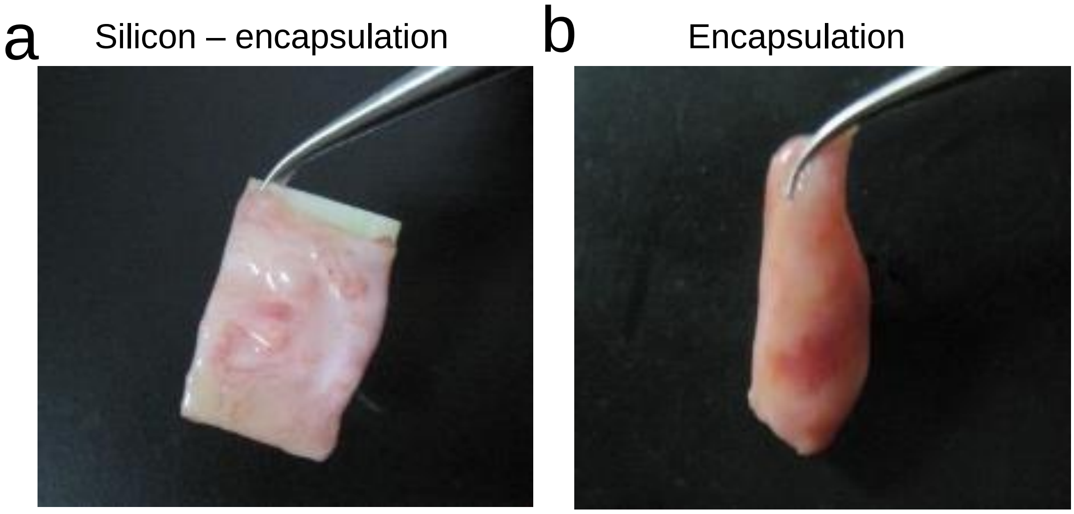

Supplementary Figure 2 Morphology of tissue capsule around a silicone sheet after 4-week subcutaneous implantation. **a**, **b**, Tissue capsule can not maintain their original shape after detachment from the silicone sheet.

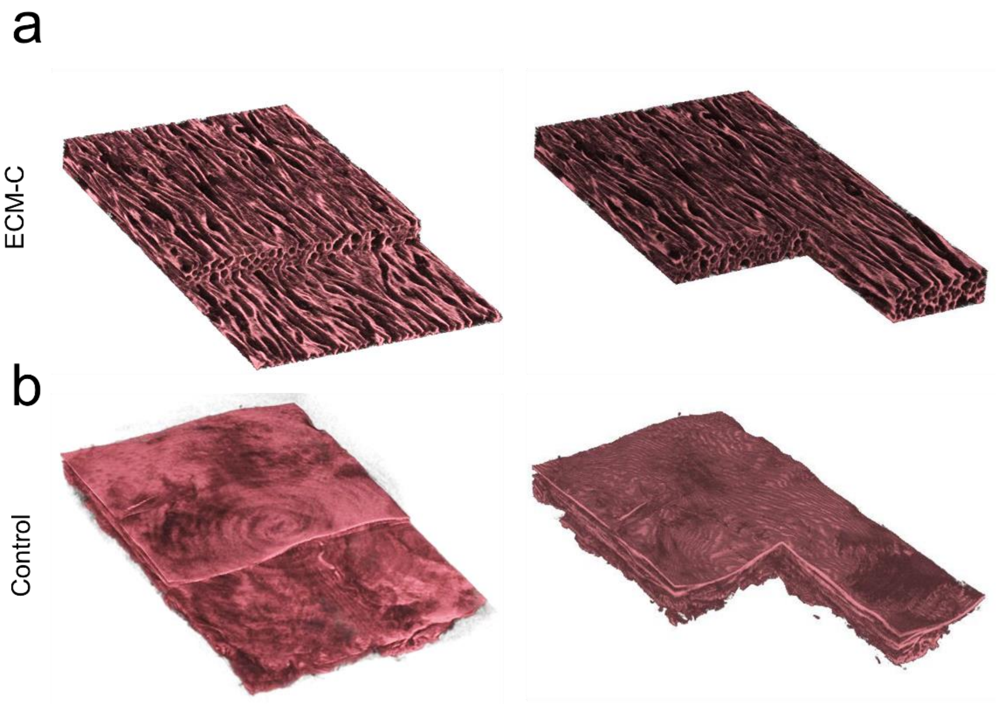

Supplementary Figure 3 Characterization of the 3D Structure of ECM-C and control scaffolds. **a**, **b**, MicroCT reconstructed image showed the macro and micro structure of ECM-C and control scaffolds at different cutting positions.

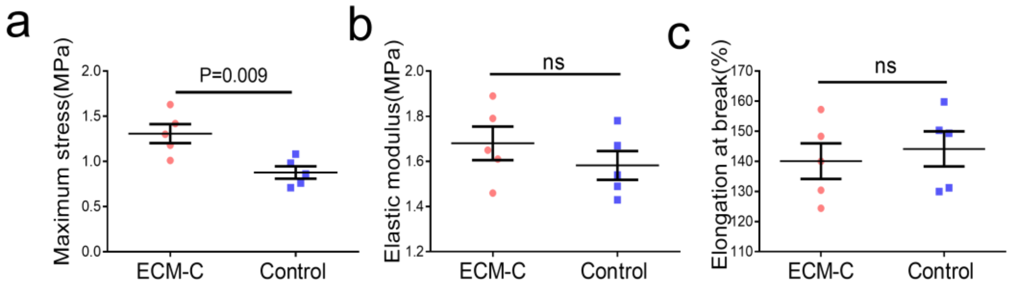

Supplementary Figure 4 Mechanical properties of the control and ECM-C scaffolds. **a, b, c**, Mechanical properties of the ECM-C and control scaffolds including maximum stress, elastic modulus and elongation at break (n = 5). Statistical analysis (ns = no significance). Bar heights and error bars represent means ± s.e.m. (t-test).

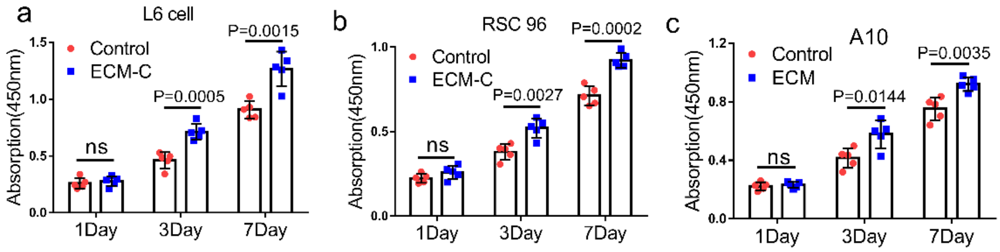

Supplementary Figure 5 Cell proliferation of L6, RSC 96 and A10 on the control and ECM-C scaffolds. **a**, **b**, **c**, CCK-8 assay showed the proliferation of L6 and RSC96 cells cultured on control and ECM-C scaffolds (n = 5). Statistical analysis (ns = no significance). Bar heights and error bars represent means ± s.e.m. (t-test).

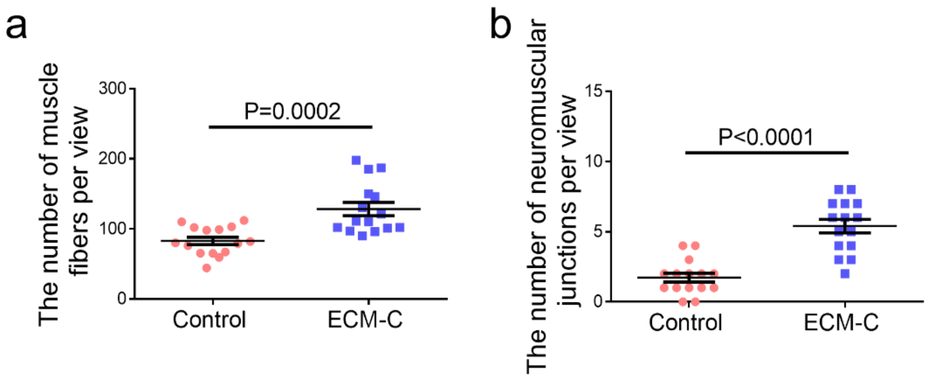

Supplementary Figure 6 Quantification of muscle fibers and neuromuscular junctions in regenerated muscles by control and ECM-C scaffolds. **a**, the number of neo-muscle fibres presented in the control and ECM-C groups; **b**, the number of neuromuscular junctions formed in the control and ECM-C groups (n = 15). n=5 rats for each group. Bar heights and error bars represent means ± s.e.m. (t-test)

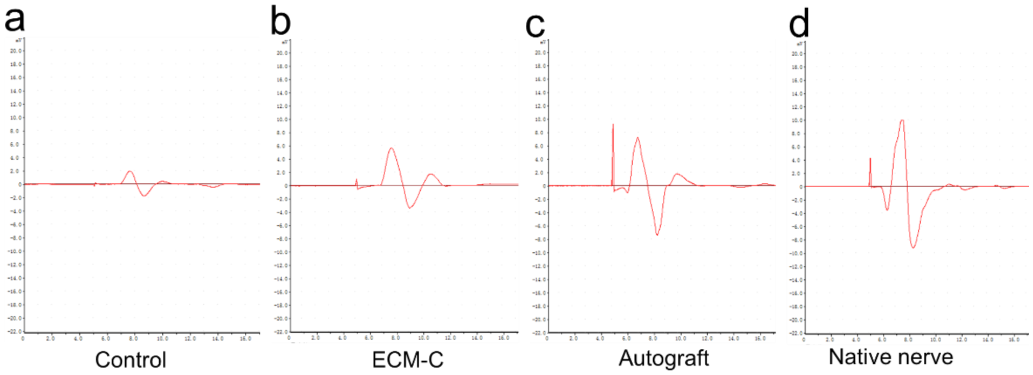

Supplementary Figure 7 Wave patterns of CMAP. **a**, **b**, **c**, **d**, Representative wave pattern images of control, ECM-C, autografts and native nerve groups.

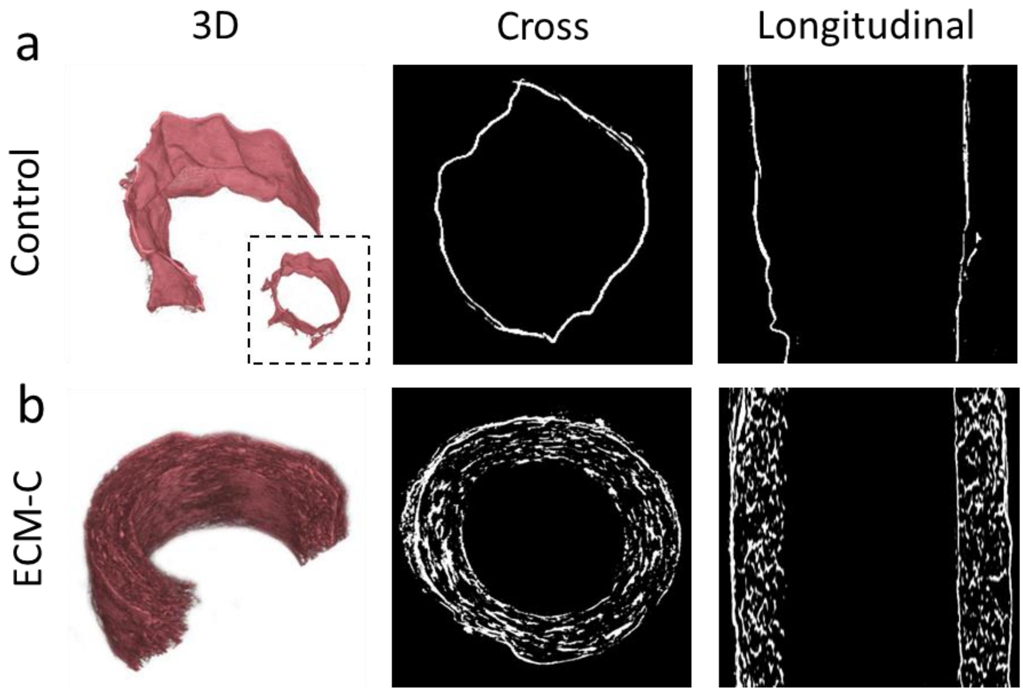

Supplementary Figure 8 Characterization of the 3D and 2D Structure of vascular ECM-C and control scaffolds. **a**, **b**, MicroCT reconstructed image showed the macro and micro structure of vascular ECM-C and control scaffolds at different cutting positions. Inset in a: macroscopic view of control scaffold.

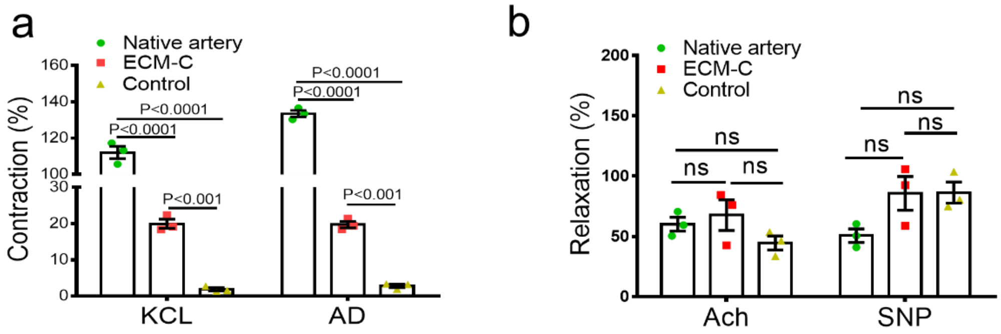

Supplementary Figure 9 Vascular function assessment in the explanted ECM-C and control scaffolds and native artery. **a**, **b**, Constriction rate in response to vasoconstrictors including KCL and adrenaline, and relaxation rate in response to the endothelial-specific activator acetylcholine (Ach) and sodium nitroprusside (SNP) vasodilators in the explanted control, ECM-C scaffolds and native artery at 3 months. The explanted scaffolds were pre-constricted with adrenaline. n=3 rats for each group. Statistical analysis (ns = no significance). Bar heights and error bars represent means ± s.e.m. (ANOVA).

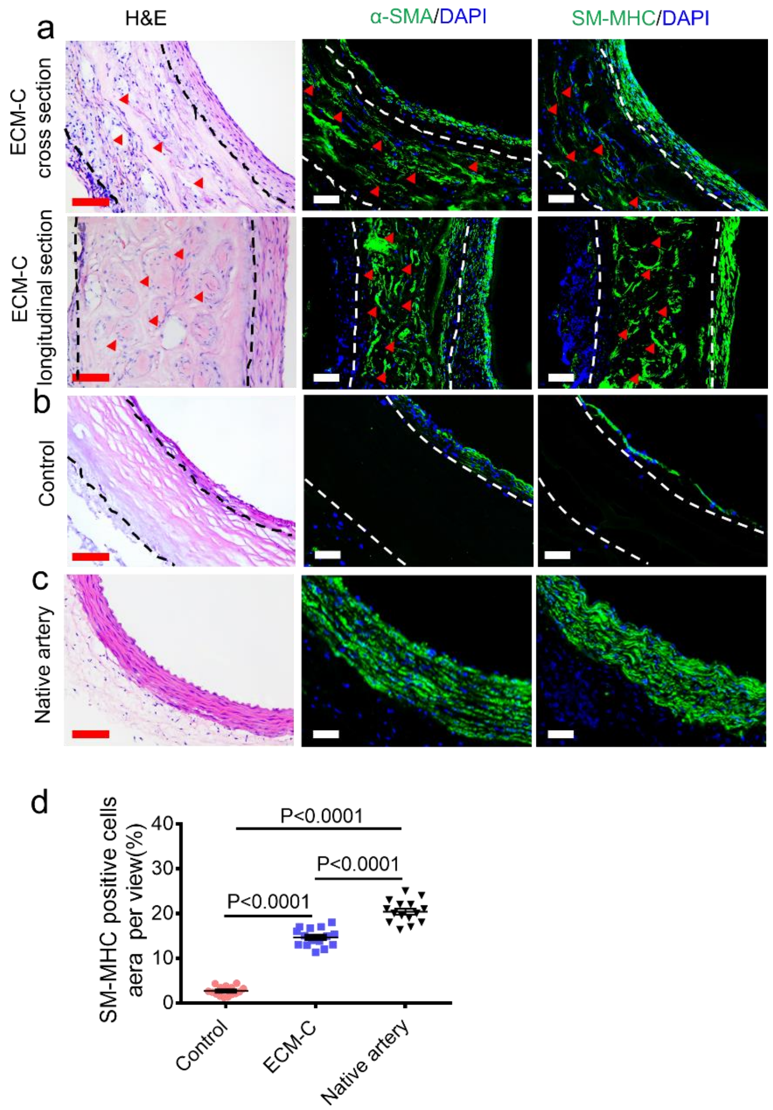

Supplementary Figure 10 VSMC regeneration and distribution in ECM-C, control scaffolds and native artery. **a, b, c,** H&E staining showing distribution of vascular cells, and immunofluorescence staining showing the distribution of α-SMA and SM-MHC positive cells (green) in control, ECM-C scaffolds and native artery. Red triangle represents the regenerated VSMC within the microchannels. Dotted lines indicate the boarder of scaffolds. **d**, SM-MHC positive cells area per view in different scaffolds (n=15). Bar heights and error bars represent means ± s.e.m. (ANOVA). Statistical analysis is shown as, ****p<0.0001. Scale bars: (c- f), 100 μm.

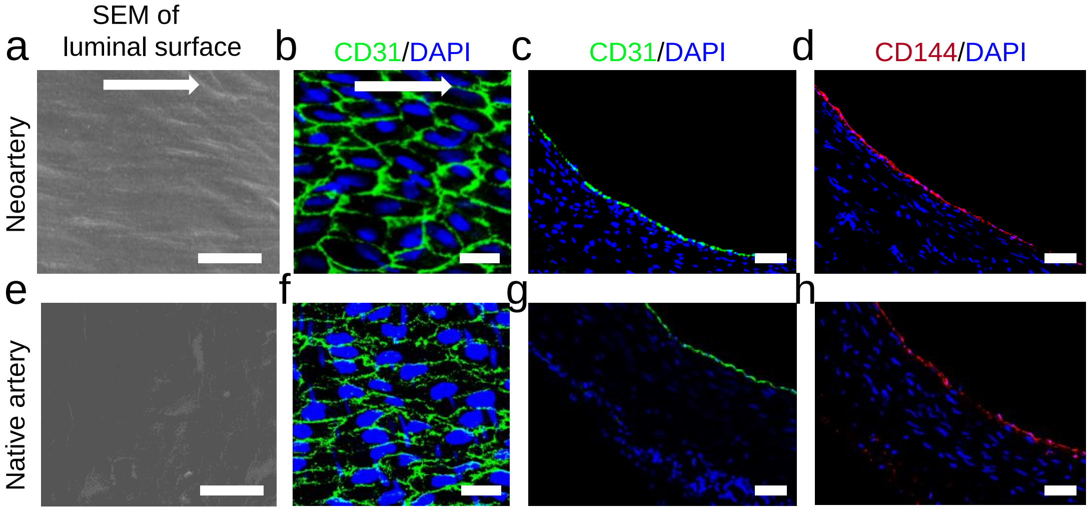

Supplementary Figure 11 Endothelialization within the neoartery and native artery at 3 months. **a**, **e**, SEM images of the luminal surface covered by cobblestone-like cells. **b**, **f**, En face view of the luminal surface stained with CD31 antibody showing endothelial cells with orientation parallel to the direction of blood flow (white arrow). **c**, **d**, **g**, **h**, The monolayer cells in the lumen surface stained for CD31 (green) and CD144 (red). The cell nuclei are stained by DAPI (blue). Scale bar: (a, e) 20 μm; (b, f,) 25 μm; (c, d, g, h) 50 μm.

Primers	Forward sequence (5'-3')	Reverse sequence (5'-3')	GenBank accession No.
MHC	GTGCCAATGACGACCTGAAGGAG	CTGGTTGATGAGGCTGGTGTTCTG	XM_017599642.1
Myogenin	GCAGTGCCATCCAGTACATTGAGC	GGAAGGTGACAGACATATCCTCCAC	NM017115.2
Krox20	CCACCTCTACTCTCCACCACCAC	TGGCGGCGATAAGAATGCTGAAG	U78102.1
NCAM	TTCAGTGACGACAGTTCGGAGC	TGCGAAGACCTTGAGGTGGAT	NM_031521.1
α-SMA	GCACCACTCCTTCTATAACG	CAGCCTGAATAGCCACATAC	NM_031004
SM-MHC	GGAGCCAAGGAGAAGATGAG	CTGAAGGACGGATGATACTACC	NM_001170600.1
NGF	TGATCGGCGTACAGGCAGA	GAGGGCTGTGTCAAGGGAAT	XM_006233053.2
GAPDH	AGGTCGGTGTGAACGGATTTG	TGTAGACCATGTAGTTGAGGTCA	NG_028301.2

Supplementary Table 1 Primer sequences of the genes for qPCR analyses
