# Supplementary material for: In vivo engineered extracellular matrix scaffolds with instructive niches for oriented tissue regeneration
Source: Nat Commun. 2019 Oct 11;10:4620. doi: 10.1038/s41467-019-12545-3 (PMC6789018; doi:10.1038/s41467-019-12545-3)
Supplement: Supplementary file 3 — Description of Additional Supplementary Files [file 41467_2019_12545_MOESM3_ESM.pdf]

Description of Additional Supplementary Files

File Name: Supplementary Movie 1
Description: Video of microCT dynamic scanning of representative membraneous ECM-C scaffolds

File Name: Supplementary Movie 2
Description: Video of microCT dynamic scanning of representative control membranous scaffolds

File Name: Supplementary Movie 3
Description: Video of microCT dynamic scanning of representative ECM-C nerve scaffolds

File Name: Supplementary Movie 4
Description: Video of microCT dynamic scanning of representative control nerve scaffolds

File Name: Supplementary Movie 5
Description: Video of microCT dynamic scanning of representative ECM-C vascular scaffolds

File Name: Supplementary Movie 6
Description: Video of microCT dynamic scanning of representative control vascular scaffolds

File Name: Supplementary Movie 7
Description: Doppler ultrasound showing synchronous pulsation of ECM-C guided neoartery.

File Name: Supplementary Movie 8
Description: Video of ECM-C guided neoartery integration with the host artery
