# Supplementary material for: In vivo engineered extracellular matrix scaffolds with instructive niches for oriented tissue regeneration
Source: Nat Commun. 2019 Oct 11;10:4620. doi: 10.1038/s41467-019-12545-3 (PMC6789018; doi:10.1038/s41467-019-12545-3)
Supplement: Supplementary file 12 — Reporting Summary [file 41467_2019_12545_MOESM12_ESM.pdf]

nature research

Corresponding author(s): Deling kong

Last updated by author(s): Aug 30, 2019

Reporting Summary

Nature Research wishes to improve the reproducibility of the work that we publish. This form provides structure for consistency and transparency in reporting. For further information on Nature Research policies, see Authors & Referees and the Editorial Policy Checklist.

Statistics

For all statistical analyses, confirm that the following items are present in the figure legend, table legend, main text, or Methods section.

n/a	Confirmed	
☐	☒	The exact sample size (*n*) for each experimental group/condition, given as a discrete number and unit of measurement
☐	☒	A statement on whether measurements were taken from distinct samples or whether the same sample was measured repeatedly
☐	☒	The statistical test(s) used AND whether they are one- or two-sided<br>*Only common tests should be described solely by name; describe more complex techniques in the Methods section.*
☐	☒	A description of all covariates tested
☐	☒	A description of any assumptions or corrections, such as tests of normality and adjustment for multiple comparisons
☐	☒	A full description of the statistical parameters including central tendency (e.g. means) or other basic estimates (e.g. regression coefficient) AND variation (e.g. standard deviation) or associated estimates of uncertainty (e.g. confidence intervals)
☐	☒	For null hypothesis testing, the test statistic (e.g. *F*, *t*, *r*) with confidence intervals, effect sizes, degrees of freedom and *P* value noted<br>*Give P values as exact values whenever suitable.*
☒	☐	For Bayesian analysis, information on the choice of priors and Markov chain Monte Carlo settings
☐	☒	For hierarchical and complex designs, identification of the appropriate level for tests and full reporting of outcomes
☐	☒	Estimates of effect sizes (e.g. Cohen's *d*, Pearson's *r*), indicating how they were calculated

Our web collection on statistics for biologists contains articles on many of the points above.

Software and code

Policy information about availability of computer code

Data collection

Mechanical testing: The properties were measured on the Instron 3345 tensile-testing machine (Norwood, Massachusetts, US)

MicroCT analysis: Bruker SkyScan Micro-CT (SkyScan 1276, Allentown, PA, USA) was used for scanning. The obtained data were reconstructed by NRecon (Version: 1.7.1.6) software. Analysis of the region of interest (ROI) was selected by CTAn (Version: 1.17.9.0) software. Porosity, orientation and anisotropy were obtained, and 3D ROI map was reconstructed.

Fibre and pore size measurements: All the scaffolds were examined by using a FEI Quanta 200 scanning electron microscope (SEM; ThermoFisher Scientific Europe NanoPort, Eindhoven, Netherlands) at an accelerating voltage of 15kV.

Cell orientation, viability and proliferation on scaffolds in vitro: Images were taken by the laser scanning confocal microscope (Leica, Germany). Live cell imaging was performed using the Infinity 3 2D array confocal scanner (Visitech Intl. Ltd. Norway) equipped with an Olympus IX81 inverted microscope system. The migration of each individual cell was tracked using the "Manual Tracking" plug-in of NIH ImageJ software to output (x, y) position coordinates during the observation period.

Real-time quantitative polymerase chain reaction (qPCR) analysis : RNA yield was determined by using a NanoDrop spectrophotometer (NanoDrop Technologies). qPCR was executed on a CFX96 Real-Time PCR System (Bio-Rad, Hercules, CA) with a SYBR Green based real-time detection method (Roche, Mannheim, Germany).

Characterization of cell infiltration, vascularization and immune response: immunofluorescence staining was performed. Slides were observed under a fluorescence microscope (Zeiss Axio Imager Z1, Germany) and images were acquired with a digital camera (AxioCam MRm, Germany)
In vivo implantation of nerve scaffolds and corresponding evaluation: The compound muscle action potentials (CMAPs) were recorded at the ipsilateral side by an 8-channel physiological signal recorder (RM-6280C, Chengdu Instrument Factory, Chengdu, China), and the peak amplitude of CMAPs was compared among groups. Regenerated nerves were harvested, fixed, and sectioned after recording the CMAPs. The TEM images of the sections were done on Transmission electron microscope (HITACHI H-600, Hitachi, Japan)

In vivo implantation of vascular scaffolds and corresponding evaluation: the patency of the implanted scaffolds was visualized using the high-resolution ultrasound (Vevo 2100 System, Visualsonics, Canada). The sectioned samples were sputter-coated with gold and observed by SEM (Quanta200, Czech Republic). The H&E images were observed under the upright microscope (Leica DM3000, Germany).

Data analysis

MicroCT analysis: CTvox (Version: 3.3.0.0) software was used for 3D image analysis and video production.
Fibre and pore size measurements: The SEM images were analyzed by Image J software v1.5.
Cell orientation, viability and proliferation on scaffolds in vitro: The images of cell orientation, viability and proliferation were analyzed by Image J software v1.5. The Euclidean distance (S) of cell migration was calculated by Chemotaxis and Migration Tool 2.0 (IBIDI, Germany) at 5-min intervals.
Real-time quantitative polymerase chain reaction (qPCR) analysis : Relative expression level of corresponding mRNA was expressed as 2-(ΔΔCT) method and normalized by housekeeping gene GAPDH.
TThe quantitative H&E, immunostaining and SEM/TEM image analysis were done by Image J software v1.5.
All statistical analyses were performed in Graphpad Prism 7. Data were presented as the mean ± standard deviation of the mean. Single comparison was made with an unpaired Student's t-test. Multiple comparisons were carried out using one-way analysis of variance (ANOVA) and Tukey's post-hoc analysis. ns = no significance

For manuscripts utilizing custom algorithms or software that are central to the research but not yet described in published literature, software must be made available to editors/reviewers. We strongly encourage code deposition in a community repository (e.g. GitHub). See the Nature Research guidelines for submitting code & software for further information.

Data

Policy information about availability of data

All manuscripts must include a data availability statement. This statement should provide the following information, where applicable:

- Accession codes, unique identifiers, or web links for publicly available datasets
- A list of figures that have associated raw data
- A description of any restrictions on data availability

The authors declare that all data supporting the findings of this study are available within the paper and its Supplementary information files or are available from the authors upon request.

Field-specific reporting

Please select the one below that is the best fit for your research. If you are not sure, read the appropriate sections before making your selection.

☒ Life sciences ☐ Behavioural & social sciences ☐ Ecological, evolutionary & environmental sciences

For a reference copy of the document with all sections, see nature.com/documents/nr-reporting-summary-flat.pdf

Life sciences study design

All studies must disclose on these points even when the disclosure is negative.

Sample size

Sample size was based on historical data which revealed statistical significance in previous experiments. All quantitative results were obtained from at least three samples and from three independent experiments.

Animal experiments: 116 male Sprague Dawley rats (male, aged 8-10 weeks with the weight range of 280-320 g) were used in this study. 40 rats were used to fabricate membranous and tubular scaffolds. 10 rats were used to evaluate the cell infiltration, vascularization and immunomodulatory properties of scaffolds, 66 rats were used for in situ implantation of muscle, nerve and vascular scaffolds. For all other experiments a minimum of n = 5 mice per group was set for obtaining numerical data.

Mechanical testing: (n = 5 samples/group)

MicroCT analysis: at least three samples and from three independent experiments

Fibre and pore size measurements: Five randomly selected SEM images were acquired for each scaffold, and images (at least 30 fibers and 30 pores) were manually measured and analyzed using ImageJ software v1.5 (NIH, Maryland, US)

Cell orientation, viability and proliferation on scaffolds in vitro: At least 40 cells were used for calculating the migration under each condition.
Real-time quantitative polymerase chain reaction (qPCR) analysis: At least five parallel samples in each group , and all data are from three independent experiments.
Characterization of cell infiltration, vascularization and immune response : The quantitative immunostaining results were obtained from at least three samples and from three independent experiments.
In vivo implantation of muscle/nerve/ vascular scaffolds and corresponding evaluation: (n = 11 animals/group)

Data exclusions

No data were excluded from the analyses.

Replication

All experiments were repeated at least three times and all attempts of replication were successful.

Randomization

Researchers were blinded to histological samples at preparation, staining and assessment phases. All samples were randomly chosen for in

Randomization	vitro or in vivo study.
Blinding	Investigators were not blinded to the treatment groups. Rats were used in this work. Blinding and randomization were applied to all experiments.

Reporting for specific materials, systems and methods

We require information from authors about some types of materials, experimental systems and methods used in many studies. Here, indicate whether each material, system or method listed is relevant to your study. If you are not sure if a list item applies to your research, read the appropriate section before selecting a response.

Materials & experimental systems

n/a	Involved in the study
☐	☒ Antibodies
☐	☒ Eukaryotic cell lines
☒	☐ Palaeontology
☐	☒ Animals and other organisms
☒	☐ Human research participants
☒	☐ Clinical data

Methods

n/a	Involved in the study
☒	☐ ChIP-seq
☒	☐ Flow cytometry
☒	☐ MRI-based neuroimaging

Antibodies

Antibodies used

Sections for vascularization: Endothelial cell staining was performed using rabbit anti-von Willebrand factor (1:200; Dako, A0082), mouse anti-CD31 (1:70; Abcam; ab64543) and CD144 primary antibody (1:100; Santa Cruz Biotechnology, sc-6458).

The smooth muscle cells were stained using mouse anti-α-SMA (1:100; Abcam; ab7817) and mouse anti-smooth muscle myosin heavy chain I (1:100; Santa Cruz Biotechnology; sc-6956) primary antibody.

ECM staining was performed using rabbit polyclonal anti-collagen I primary antibody (1:200; Abcam; ab34710), rabbit polyclonal anti-collagen III primary antibody (1:200; Abcam; ab7778), rabbit polyclonal anti-elastin (1:200; Abcam; ab21610) primary antibody, anti-laminin primary antibody (1:200; Abcam; ab11575) for collagen I, collagen III, elastin, laminin respectively.

Sections for immuno-response: To identify macrophage phenotype, immunofluorescence staining was performed with the following antibodies: CD68 (1:100, Abcam, ab31630), a general macrophage marker; CD206 (1:200, Abcam, ab64693), a marker for M2-like macrophages and inducible nitric oxide synthase (iNOS) (1:200, Abcam, ab15323) as a marker for M1-like macrophages

Sections for nerve tissue: S100 (1:200; Abcam; ab52642) and NF09 antibodies (1:200; Abcam; ab7794).

Alexa Fluor 488 goat anti-rabbit IgG (1:200; Invitrogen,) and Alexa Fluor 594 goat anti-mouse IgG (1:200; Invitrogen) were used as the secondary antibodies, respectively.

Cytoskeleton organization was visualized by fluorescently staining with phalloidin-AlexaFluor 488 (Sigma-Aldrich) and DAPI (Sigma-Aldrich).
DAPI staining was used to evaluate the cell infiltration

Validation

Positive and negative controls of specific-binding (for each of the florescent labeled targets) were included in each experiment.
Antibodies were used at the dilutions recommended by the manufacturer and validated using appropriate positive (stimulated cells) and negative (unstimulated cells) controls.
Every antibody was previously validated by the manufacturer; citations are available on the manufacturer's website.

Eukaryotic cell lines

Policy information about cell lines

Cell line source(s)	Rat Schwann cell line RSC96 , Rat skeletal muscle cell line L6 and Vascular smooth muscle cell line A10 cell were all obtained from American type culture collection (ATCC)
Authentication	Each cell line we used was morphologically confirmed according to the information provided by ATCC.
Mycoplasma contamination	All cell lines were tested for mycoplasma contamination. No mycoplasma contamination was found.
Commonly misidentified lines (See ICLAC register)	NO

Animals and other organisms

Policy information about studies involving animals; ARRIVE guidelines recommended for reporting animal research

Laboratory animals	116 male Sprague Dawley rats (male, aged 8-10 weeks with the weight range of 280-320 g) were used in this study. All the animals were purchased from the Laboratory Animal Centre of the Academy of Military Medical Sciences (Beijing, China).
Wild animals	NO
Field-collected samples	NO
Ethics oversight	Animal experiments were approved by the Animal Experiments Ethical Committee of Nankai University and complied with the Guide for Care and Use of Laboratory Animals.

Note that full information on the approval of the study protocol must also be provided in the manuscript.
